# Development of a Ready-to-Use-Type RNA Vaccine Carrier Based on an Intracellular Environment-Responsive Lipid-like Material with Immune-Activating Vitamin E Scaffolds

**DOI:** 10.3390/pharmaceutics15122702

**Published:** 2023-11-29

**Authors:** Jessica Anindita, Hiroki Tanaka, Ryotaro Oyama, Shinya Hagiwara, Daiki Shirane, Sakura Taneichi, Yuta Nakai, Kota Tange, Hiroto Hatakeyama, Yu Sakurai, Hidetaka Akita

**Affiliations:** 1 Laboratory of DDS Design and Drug Disposition, Graduate School of Pharmaceutical Sciences, Chiba University, 1-8-1 Inohana, Chuo-ku, Chiba 260-0856, Japan; 2 Laboratory of DDS Design and Drug Disposition, Graduate School of Pharmaceutical Sciences, Tohoku University, 6-3 Aoba, Aramaki, Aoba-ku, Sendai 980-8578, Japan; 3 Life Science Research Laboratory, NOF CORPORATION, 3-3 Chidori-cho, Kawasaki-ku, Kawasaki 210-0865, Japan

**Keywords:** lipid nanoparticle, freeze-drying, mRNA vaccine

## Abstract

Because of its efficient and robust gene transfer capability, messenger RNA (mRNA) has become a promising tool in various research fields. The lipid nanoparticle (LNP) is considered to be a fundamental technology for an mRNA delivery system and has been used extensively for the development of RNA vaccines against SARS-CoV-2. We recently developed ssPalm, an environmentally responsive lipid-like material, as a component of LNP for mRNA delivery. In this study, a self-degradable unit (phenyl ester) that confers high transfection activity and an immune stimulating unit (vitamin E scaffold) for high immune activation were combined to design a material, namely, ssPalmE-Phe-P4C2, for vaccine use. To design a simple and user-friendly form of an RNA vaccine based on this material, a freeze-drying-based preparation method for producing a ready-to-use-type LNP (LNP(RtoU)) was used to prepare the LNP_ssPalmE-Phe_. The optimization of the preparation method and the lipid composition of the LNP_ssPalmE-Phe_(RtoU) revealed that dioleoyl-sn-glycero phosphatidylethanolamine (DOPE) was a suitable helper lipid for achieving a high vaccination activity of the LNP_ssPalmE-Phe_(RtoU). Other findings indicated that to maintain particle properties and vaccination activity, a 40% cholesterol content was necessary. A single administration of the LNP_ssPalmE-Phe_(RtoU) that contained mRNA-encoding Ovalbumin (mOVA-LNP_ssPalmE-Phe_(RtoU)) demonstrated a significant suppression of tumor progression in a tumor-bearing mouse OVA-expressing cell line (E.G7-OVA). In summary, the LNP_ssPalmE-Phe_(RtoU) is an easy-to-handle drug delivery system (DDS) for delivering mRNA antigens in immunotherapy.

## 1. Introduction

Nucleic-acid-based vaccines are based on the use of plasmid DNA (pDNA) or messenger RNA (mRNA) as a source of antigens. Upon the administration of such a vaccine, these genetic materials need to be delivered into the nucleus/cytoplasm, their site of action, where they will be transcribed/translated into antigen proteins. Since the mRNA can function in the cytoplasm and nuclear localization is not required, the mRNA can provide efficient and robust gene transfer, even in non-dividing cells [1,2,3]. On the other hand, since mRNA is highly susceptible to enzymatic degradation in extracellular fluid, the development of suitable delivery technology is a prerequisite for its clinical application [3,4]. One of the more promising carriers for RNA delivery is lipid nanoparticles (LNPs), which contain an ionizable lipid as the main component. In 2018, ONPATTRO^®^ (Alnylam Pharmaceutical, Cambridge, MA, USA), an LNP-based therapeutic using small interfering RNA (siRNA), became the first ever approved RNA interference (RNAi) therapeutic for the treatment of hereditary transthyretin-mediated amyloidosis [5,6,7,8,9]. Moreover, the approval of RNA vaccines (mRNA-LNPs) against severe acute respiratory syndrome coronavirus 2 (SARS-CoV-2) [10,11,12,13,14] further demonstrated the versatility of the LNP as an mRNA carrier.

We have been developing LNPs that are composed of a series of ionizable lipids, referred to as SS-cleavable and pH-activated lipid-like materials (ssPalm). The ssPalm(s) respond to the difference in extracellular and intracellular environments based on their dual-sensing motifs: tertiary amines and a disulfide bond. After cellular uptake, the neutrally charged LNPs_ssPalm_ are sorted into endosomes. When arriving in the endosome, the LNPs are exposed to the acidic environment in the endosomal compartment (pH 6.5–5.5) and develop positive charges on their surface through the protonation of tertiary amines. The positively charged LNPs then cause endosomal membrane disruption/destabilization, which results in the endosomal escape of their cargo. Following this endosomal escape event, the disulfide bond in LNPs_ssPalm_ will be cleaved by glutathione in the reductive environment of the cytoplasm. This then triggers the decapsulation of the mRNA cargoes, releasing them into the cytoplasm [15,16,17].

As additional modifications of the ionizable lipids, we have focused on the hydrophobic scaffold and the linker between the amine and the scaffolds. Derivatives of ssPalms can be developed by changing their hydrophobic scaffolds. Changing the hydrophobic scaffolds from fatty acids such as myristic acid (ssPalmM) to retinoic acid (vitamin A; ssPalmA) allowed the intracellular trafficking of the nucleic acid cargo to be controlled [15]. Types of ssPalm with vitamin E scaffolds (ssPalmE) have become a focus of vaccine development since they are equipped with both gene expression activity and immune activation properties [16]. The vitamin E scaffolds were assumed to be suitable for cancer vaccines because they can efficiently activate cell-mediated immunity via the type I interferon pathway [18]. In addition to the modification of hydrophobic scaffolds, the incorporation of a self-degradable phenyl ester moiety can be used to improve the gene transfer activity of the mRNA-LNP [15,17]. The cleavage of disulfide bonds by reducing agents in the cytoplasm elevates the concentration of hydrophobic thiols in the particle. Within those limited intraparticle spaces, the concentrated thiols attack the phenyl ester and trigger a nucleophilic substitution reaction. This self-degradation reaction is irreversible, leading to the more efficient collapse of particles, which further enhances the release of the nucleic acid cargo [15,17]. In this study, a phenyl ester moiety (for achieving high transfection activity) and vitamin E scaffolds (for promoting immune activation) were combined to design ssPalmE-Phe-P4C2 for RNA vaccine development.

LNPs are generally prepared using the alcohol dilution method. In this method, the lipids and nucleic acids spontaneously form a complex due to the electrostatic interactions between the positively charged amine groups and the negatively charged nucleic acids, as well as the hydrophobic interactions between the hydrophobic moieties of lipids [19]. However, we recently reported that the post-encapsulation of mRNA into a pre-formed LNP is also applicable for use in preparing an mRNA-LNP [20]. Therefore, it is possible to encapsulate a desired mRNA into an mRNA-LNP by using pre-formed ready-to-use (RtoU) types of LNPs. Since the LNP(RtoU) can be used simply by rehydration and incubation, only a typical temperature-controlling device (i.e., block incubator, water bath, or thermal cycler) is needed for use. The development of an LNP(RtoU) using ssPalmE-Phe-P4C2 (LNP_ssPalmE-Phe_(RtoU)) would produce a convenient form of an mRNA vaccine that offers handling practicality and vaccination efficacy. In this study, we investigated the potency of ssPalmE-Phe-P4C2 as an RNA vaccine and optimized the conditions needed for the preparation of the LNP_ssPalmE-Phe_(RtoU). The ability to induce cytotoxic T lymphocyte (CTL) activation and antitumor activity against an E.G7-OVA tumor model was also investigated.

## 2. Materials and Methods

### 2.1. Animals

C57BL/6J mice (female, 6 weeks) were purchased from Japan SLC, Inc. (Shizuoka, Japan). Protocols for the animal experiments were reviewed and approved by the Chiba University Animal Care Committee following the “Guide for Care and Use of Laboratory Animals”.

### 2.2. Materials

A detailed list of supplier information, including the item numbers of all the reagents used in this study, is listed in Supporting Information (Table S1). The ssPalmE-Phe-P4C2, ssPalmE-P4C2 (Product# COATSOME^®^ SS-EC), 1,2-dioleoyl-*sn*-glycero-3-phosphatidylcholine (DOPC, Product# COATSOME^®^ MC-8181), and 1-(Monomethoxy polyethyleneglycol2000)2,3-dimyristoylglycerol (DMG-PEG_2000_, Product# SUNBRIGHT^®^ GM-020) were supplied by NOF CORPORATION (Tokyo, Japan). The 1,2-dioleoyl-*sn*-glycero-3-phosphoethanolamine (DOPE, Product# 18:1 (△9-Cis) PE (DOPE)) was purchased from Avanti^®^ Polar Lipids (Alabaster, AL, USA). Cholesterol was purchased from SIGMA Aldrich (St. Louis, MO, USA). The mRNA-encoding gene (Firefly Luciferase or Ovalbumin (OVA)) was prepared through the in vitro transcription reaction described in the following section. The Quant-IT™ RiboGreen^®^ RNA reagent was purchased from Thermo Fisher Scientific (Waltham, MA, USA). All other reagents and chemicals were commercially available and used without further purification.

### 2.3. Cell Culture

Bone-marrow-derived dendritic cell (BMDC): Murine BMDCs were isolated from the femurs of C57BL/6J mice. Bone marrow cells were cultured over 6 h in Roswell Park Memorial Institute (RPMI)-1640 medium (#R8758, SIGMA Aldrich, St. Louis, MO, USA) supplemented with 10% (*v*/*v*) Fetal Calf Serum (FCS, #SH30910.03, Hyclone, Logan, UT, USA), 50 µM of 2-mercaptoethanol (#21985023, Thermo Fisher Scientific, Waltham, MA, USA), 10 mM of HEPES buffer (#17557-94, nacalai tesque, Kyoto, Japan), 1 mM of sodium pyruvate (#06977-34, nacalai tesque, Kyoto, Japan), and 100 U/mL of penicillin/streptomycin (#26253-84, nacalai tesque, Kyoto, Japan). The non-adherent cells were harvested and cultured in another dish with the same medium with an additional 10 mg/mL of Recombinant Mouse GM-CSF (#415-ML-050, R&D Systems, Minneapolis, MN, USA). Non-adherent cells were further removed on Days 2 and 4. The remaining adherent cells were cultured in a fresh culture medium containing 10 mg/mL of GM-CSF. On Day 6, the non-adherent cells were used in the experiments as immature BMDCs [16].

RAW 264.7 cell: The RAW 264.7 cells, a murine macrophage cell line, were cultured in RPMI-1640 medium (#R8758, SIGMA Aldrich, St. Louis, MO, USA) supplemented with 10% (*v*/*v*) Fetal Bovine Serum (FBS, #10270, Gibco, New York, USA) and 100 U/mL of penicillin/streptomycin (#26253-84, nacalai tesque, Kyoto, Japan). The adherent cells were peeled from the bottom of the dish with 0.25% Trypsin/EDTA (#32777-15, nacalai tesque, Kyoto, Japan) and cultured in another dish with a fresh medium. These cells were passaged every 2 days. The cells were used in the experiments after the third cell passage.

E.G7-OVA cell: E.G7-OVA cells, a murine lymphoma cell line EL4-expressing OVA, were purchased from the American Type Culture Collection (Manassas, VA, USA). E.G7-OVA cells were cultured in RPMI-1640 medium (#R8758, SIGMA Aldrich, St. Louis, MO, USA) supplemented with 10% (*v*/*v*) FCS (#SH30910.03, Hyclone, Logan, UT, USA), 50 µM of 2-mercaptoethanol (#21985023, Thermo Fisher Scientific, Waltham, MA, USA), 10 mM of HEPES buffer (#17557-94, nacalai tesque, Kyoto, Japan), 1 mM of sodium pyruvate (#06977-34, nacalai tesque, Kyoto Japan), 400 µg/mL of G418 Sulfate (#074-05963, FUJIFILM Wako Pure Chemical Corporation, Tokyo, Japan), and 100 U/mL of penicillin/streptomycin (#26253-84, nacalai tesque, Kyoto, Japan). The cells were collected and cultured in another dish with a fresh medium every 2 days (cell passage). The cells were used in the experiments after the third cell passage [16].

### 2.4. In Vitro mRNA Transcription (IVT-mRNA)

The pcDNA3.1 vector was used as a coding template for Luciferase (Luc) and ovalbumin (OVA). The pcDNA3.1-Luc or pcDNA3.1-OVA were linearized with the restriction enzymes BspEI (#R0540S, New England Biolabs, Ipswich, MA, USA) or XhoI (#R0146S, New England Biolabs, Ipswich, MA, USA), respectively. After phenol-chloroform extraction and ethanol precipitation, the linearized pDNA was transcribed into mRNA with a mMESSAGE mMACHINE T7 ULTRA Transcription kit (#AM1345, Invitrogen, Waltham, MA, USA) following the manufacturer’s instructions. The transcribed mRNA was dissolved in THE RNA Storage Solution (#AM7000, Invitrogen, Waltham, MA, USA) and stored at −80 °C. The concentrations of linearized pDNA and transcribed mRNA were measured with a microvolume UV-Vis spectrometer (NanoDrop™ One, Thermo Fisher Scientific, Waltham, MA, USA).

### 2.5. Preparation of Microfluidic LNPs_ssPalm_

The lipid composition was ssPalmE-P4C2 (or ssPalmE-Phe-P4C2)/DOPE (or DOPC)/Chol = 60/30/10 with additional DMG-PEG_2000_ (3 mol% of total lipid). The total lipid amount was 394.5 nmol to encapsulate 3 µg of mRNA (lipid/mRNA: 131.5 nmol/µg). The lipid mixtures were dissolved in 99.5% ethanol to a concentration of 1.32 mM. The mLuc or mOVA were diluted in 20 mM of Malic acid buffer (30 mM NaCl, pH 3.0) to a concentration of 0.0033 µg/µL. The lipid mixtures in the ethanol and mRNA solutions were set to their respective syringes in the NanoAssemblr™ Benchtop device (Precision Nanosystems, Vancouver, Canada with the settings of flow rate = 2.5 mL/min, flow rate ratio (buffer:lipid) = 3:1, and total volume = 1.5 mL. The LNPs were recovered and diluted with 20 mM of MES buffer (pH 5.5). The external solution of the LNPs was replaced with D-PBS (−) through ultrafiltration using Amicon Ultra-4-100K Centrifugal Units (#UFC810096, # UFC910096, Merck, Rahway, NJ, USA). The LNPs were then diluted to an adequate volume with D-PBS (−).

### 2.6. Preparation of LNPs_ssPalmE-Phe_ (RtoU)

The lipid composition was ssPalmE-Phe-P4C2/DOPE (or DOPC)/Chol = 52.5/7.5/40 with additional DMG-PEG_2000_ (3 mol% of total lipid) [17]. The amount of total lipid was 100 nmol, encapsulating 0.5 µg of mRNA (lipid/mRNA: 200 nmol/µg). The lipid mixtures were dissolved in 90% t-butanol to a concentration of 8.0 mM. The lipid mixtures in t-butanol and buffer solution, containing 40 mM MES buffer (pH 5.0, salt-free) with a 744 mg/mL solution of sucrose (in equal volume), were set to their respective syringes in the NanoAssemblr™ Benchtop device (Precision Nanosystems, Vancouver, Canada) with the settings of flow rate = 1.0 mL/min, flow rate ratio (buffer:lipid) = 3:1, and total volume = 0.52 mL. An equal volume of 320 mg/mL of sucrose solution (#30404-45, nacalai tesque, Kyoto, Japan) was added (final concentration of sucrose in the LNP solution = 320 mg/mL). The LNP solution was frozen in liquid nitrogen and transferred to the freeze-dryer (FDU-2110, EYELA, Tokyo, Japan). The drying sequence of the freeze-dryer was set to 9 segments: −40 °C (±7 °C, 40 min), −40 °C (vacuum start, 20 min), −30 °C (1 h), −20 °C (1 h), −10 °C (1 h), 0 °C (1 h), 10 °C (1 h), 20 °C (1 h), and 30 °C (3 h). The freeze-dried LNPs were recovered, and their physical appearances were inspected for visible cracks, dents, or collapses in front of a black background [21]. The dry LNPs were reconstituted with a water solution containing 0.5 µg of either mLuc or mOVA (0.0025 µg/µL). The solution was mixed well, followed by heating at 95 °C for 5 min, then allowed to cool to room temperature (RT) for approximately 10 min before use in the experiments/analysis.

### 2.7. Characterization of mRNA-LNPs

The particle size, polydispersity index (PdI), and zeta-potential of the LNPs were measured via dynamic light scattering (Zetasizer Nano ZS, Malvern Panalytical, Malvern, UK). The recovery ratio and encapsulation efficiency of the mRNA were evaluated with RiboGreen^®^ assay. Solutions containing Quant-iT™ RiboGreen^®^ RNA reagent (#R11491, Invitrogen, Waltham, MA, USA) with or without 10% (*v*/*v*) TritonX-100 (#168-11805, FUJIFILM Wako Pure Chemical Corporation, Tokyo, Japan), in respective 1:8 ratios were prepared in D-PBS (−): Triton [+] (contained both RiboGreen^®^ and TritonX-100) and Triton [−] (no TritonX-100, only RiboGreen^®^). The mRNA-LNPs, corresponding to 50 ng of mRNA in 50 µL of D-PBS (−), were prepared twice and each mixed with an equal volume of the Triton [+] and Triton [−] solution in a 96-well black microplate. A calibration curve was prepared through sequential dilution from 0 to 2000 ng/mL of mRNA. The plate was incubated for 5 min with shaking at 500 rpm in a shaking incubator. The fluorescence intensities were analyzed with a plate reader (Infinite M200 PRO, TECAN, Männedorf, Switzerland) set with emission and excitation waves of 484 nm and 535 nm, respectively. The recovery ratio was calculated from the total mRNA (quantified by Triton [+] addition) and mRNA concentration input (standard curve). The encapsulation efficiency was calculated as the ratio of the total mRNA concentration (quantified by Triton [+]) to the non-encapsulated mRNA concentration (quantified by Triton [−] addition).

### 2.8. Evaluation of In Vitro Gene Expression Efficiency

BMDCs (8 × 10^5^ cells/mL) and RAW 264.7 cells (2 × 10^5^ cells/mL) seeded in a 12-well plate were transfected with mLuc-LNPs in D-PBS (−) at a dose of 0.8 µg of mRNA. In the case of RAW 264.7 cells, the transfection was demonstrated after pre-incubation for 24 h. After transfection, the BMDCs and RAW 264.7 cells were incubated at 37 °C with 5% CO_2_ for 5 h. The BMDCs and RAW 264.7 cell suspensions were collected, washed with D-PBS (−), and then solubilized with Reporter Lysis Buffer (#E397A, Promega, Madison, WI, USA). The cell lysates were added with a Nano-Glo^®^ Luciferase Assay System (#E1501, Promega), and the luminescence intensities were quantified with a luminometer (GloMax^®^ 20/20 Luminometer, Promega, Madison, WI, USA). The protein amount in the cell lysates was determined using the Bicinchoninic acid (BCA) Protein Assay kit (#T9300A, Takara Bio Inc., Kusatsu, Shiga, Japan) according to the manufacturer’s instructions. Luciferase activity was represented as a relative light unit (RLU/mg protein), calculated by dividing the luminescence intensity by the protein amount [16].

### 2.9. Evaluation of In Vivo Gene Expression Efficiency (IVIS Imaging)

The mLuc-LNPs in D-PBS (−) were administered subcutaneously (back of neck) to C57BL/6J mice at a dose of 1.0 µg of mRNA under anesthetized conditions. The neck region of mice was shaved in advance. Six hours later, D-Luciferin potassium (#126-05116, FUJIFILM Wako Pure Chemical Corporation, Tokyo, Japan) in D-PBS (−) (3 mg/200 µL/mouse) was administered intraperitoneally. After 30 min had passed, the luminescence intensities were measured with an In Vivo Imaging System (IVIS^®^ Lumina II, Caliper Life Sciences, Waltham, MA, USA).

### 2.10. In Vivo Cytotoxic T Lymphocyte (CTL) Assay

The in vivo CTL assay was performed as described previously [16]. The mOVA-LNPs in D-PBS (−) were injected subcutaneously (back of neck) to C57BL/6J mice at a dose of 0.05 or 0.1 µg of mRNA under anesthetized conditions. Seven days after the LNP administration (immunization), the non-treated (NT) mice were sacrificed, and their spleens were collected into a dish containing RPMI-1640 medium (#R8758, SIGMA Aldrich, St. Louis, MO, USA) supplemented with 10% (*v*/*v*) FCS (#SH30910.03, Hyclone, Logan, UT, USA), 50 µM of 2-mercaptoethanol (#21985023, Thermo Fisher Scientific, Waltham, MA, USA), 10 mM of HEPES buffer (#17557-94, nacalai tesque, Kyoto, Japan), 1 mM of sodium pyruvate (#06977-34, nacalai tesque, Kyoto, Japan), and 100 U/mL of penicillin/streptomycin (#26253-84, nacalai tesque, Kyoto, Japan). The cell suspension of the splenocytes was filtered through a 40 µm cell strainer and resuspended in Red Blood Cell Lysing Buffer (#R7757-100ML, SIGMA Aldrich, St. Louis, MO, USA). The cell suspension was washed and resuspended in a fresh medium, then equally divided into two suspensions: CFSE^high^- and CFSE^low^-labelled. Each cell suspension was resuspended in a 1.0 × 10^7^ cells/mL cell concentration. The OVA H-2K^b^ cytotoxic T-lymphocyte epitope peptide (SIINFEKL, OVA_257–264_) in DMSO was added to the CFSE^high^-labelled cells (1/400 of the suspension volume). Both cell suspensions were incubated at 37 °C with 5% CO_2_ for 1 h. Each cell suspension was adjusted to a 3.0 × 10^7^ cells/mL cell concentration in D-PBS (−). The CFSE^high^ (5.0 µM) and CFSE^low^ (0.5 µM) (Cellstain CFSE, #C375, Dojindo Laboratories, Kumamoto, Japan) cells were added to their respective cell suspension tubes and incubated in a 37 °C water bath for 10 min under a light-shielding condition. Each cell suspension was washed repeatedly with a fresh medium and D-PBS (−), then resuspended in 5.0 × 10^7^ cells/mL cell concentration in D-PBS (−). The cells were administered intravenously into the immunized mice with equal volumes (ratio 1:1) of CFSE^high^- and CFSE^low^-labelled splenocytes. Twenty hours after administration, the spleens were collected from the immunized mice, and the splenocytes were suspended into single-cell suspensions in FACS buffer (0.5% Bovine Serum Albumin (#01860-07, nacalai tesque, Kyoto, Japan) and 0.1% NaN_3_ (#194-01275, FUJIFILM Wako Pure Chemical Corporation, Tokyo, Japan) in D-PBS (−)). The number of CFSE-labeled cells (CFSE^high^ and CFSE^low^) was quantified with a flow cytometer (NovoCyte Flow Cytometer, Agilent, Santa Clara, CA, USA). The CTL activity was represented by the degree of cell lysis, calculated as the ratio of the number of CFSE^high^-labelled cells to CFSE^low^-labelled cells.

### 2.11. Therapeutic Anti-Tumor Response against E.G7-OVA

E.G7-OVA cells (8.0 × 10^7^ cells/40 µL) suspended in D-PBS (−) were inoculated subcutaneously on the left flank of mice in anesthetized conditions. After the tumor grew to ≥100 mm^3^ (approximately after 7–9 days), the mice were injected subcutaneously (back of neck) with mOVA-LNPs (0.5 µg of mRNA) in D-PBS (−). Tumor sizes were measured at 3-day intervals with the calculation formula: {long axis} × {short axis}^2^ × 0.52 [16]. The endpoint of tumor measurement was set to 1000 mm^3^.

## 3. Results

### 3.1. Transfection Efficiency and Immune Activity of LNPs

**Figure 1:**
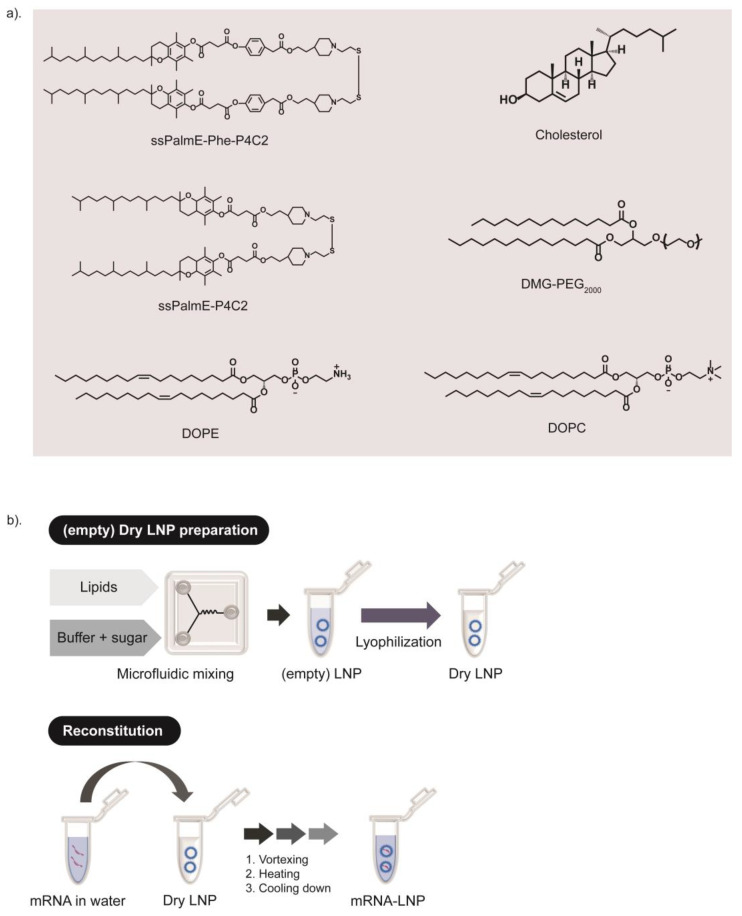
Chemical structure of lipid components and schematic illustration of the experimental design. (**a**) The molecular structures of ssPalmE-Phe-P4C2, ssPalmE-P4C2, DOPE, DOPC, cholesterol, and DMG-PEG_2000_ are shown. (**b**) Experimental design of LNP_ssPalmE-Phe_(RtoU) preparation; the empty LNPs were prepared through microfluidic mixing and then lyophilized to produce dry empty LNPs. The mRNA-encapsulated LNPs could be obtained via the reconstitution (rehydration) of the dry LNPs with the mRNA solution in water.

**Figure 2:**
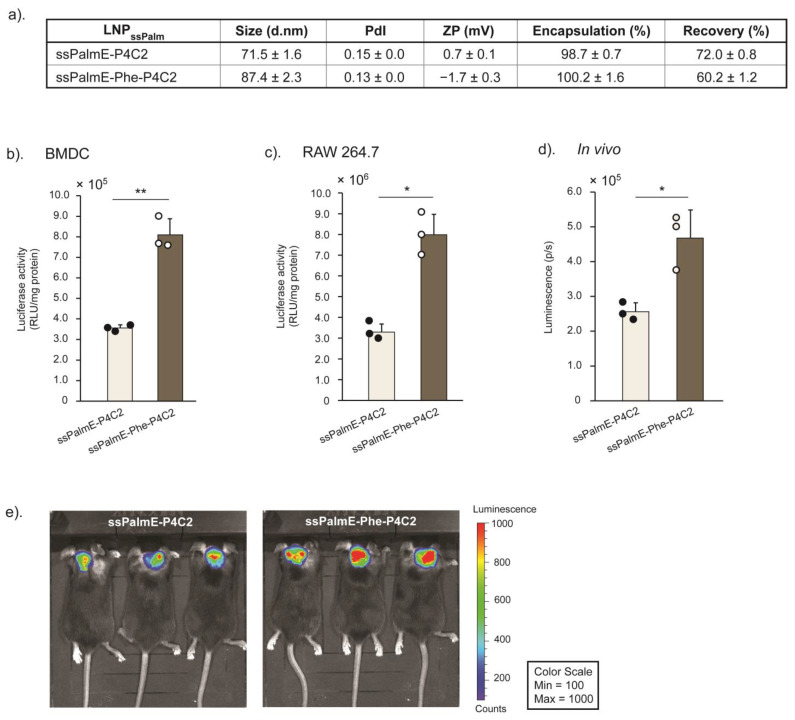
In vitro and in vivo gene expressions of LNPs_ssPalmE_ and LNPs_ssPalmE-Phe_ prepared with a microfluidic device. (**a**) Physicochemical properties of LNPs_ssPalmE_ and LNPs_ssPalmE-Phe_. In vitro gene expression after transfection of mLuc-LNPs at a dose of 0.8 µg of mRNA in (**b**) BMDCs (8.0 × 10^5^ cells) and (**c**) RAW 264.7 cells (2.0 × 10^5^ cells). Luciferase activity is represented as a relative light unit (RLU/mg protein), calculated from the luminescence intensities and protein content of the cell lysates. (**d**,**e**) Quantification and imaging results of the in vivo mLuc-LNP gene expression in C57BL/6J mice through immunization (s.c.) of 1.0 µg of mRNA. Six hours after administration, D-luciferin potassium (3 mg/200 µL/mouse) was administered (i.p.), and the luminescence intensities were measured using an IVIS device. The scatter graph represents the individual value; the bar graph represents the mean with SD (*n* = 3); * *p* < 0.05; ** *p* < 0.01 (two-tail unpaired *t*-test). The measurements in the in vitro experiments were conducted in triplicate.

The ssPalmE-Phe-P4C2 (Figure 1a) was developed by introducing a phenyl ester moiety into ssPalmE-P4C2, a derivative of ssPalm with a vitamin E scaffold [15,17]. Helper lipids, such as 1,2-dioleoyl-*sn*-glycero-3-phosphatidylcholine (DOPC) or 1,2-dioleoyl-*sn*-glycero-3-phosphoethanolamine (DOPE), cholesterol, and 1-(Monomethoxy polyethyleneglycol2000)2,3-dimyristoylglycerol (DMG-PEG_2000_) (Figure 1a), were incorporated to stabilize the LNP formulation. To investigate the effects of the phenyl ester group that was inserted into the structure, the transfection efficiency and immune activation ability of the LNPs containing the ssPalmE-Phe-P4C2 (LNPs_ssPalmE-Phe_) were compared with those containing the ssPalmE-P4C2 (LNPs_ssPalmE_). The LNP_ssPalmE-Phe_- and LNP_ssPalmE_-containing luciferase mRNAs (mLuc-LNPs) were prepared by means of a microfluidic mixer. The composition of the LNPs was ssPalm/DOPE/Chol = 60/30/10 with an additional 3% DMG-PEG_2000_ [17]. The properties of both the mLuc-LNPs_ssPalmE-Phe_ and mLuc-LNPs_ssPalmE_ were similar, with sizes of around 70–90 nm, an acceptable polydispersity index (PdI) (<0.200), and a neutral surface charge (0 to −2 mV). Both types of LNPs had a high mRNA encapsulation rate (>90%) and comparable recovery rates (60–75%) (Figure 2a).

Both an in vitro and in vivo quantifications of gene expression were performed to evaluate the effect of the phenyl ester group on transfection efficiency. An in vitro luciferase assay was performed using bone-marrow-derived dendritic cells (BMDCs) (Figure 2b) and RAW 264.7 cells of a murine macrophage/monocyte cell line (Figure 2c). Luciferase activity was evaluated 6 h after the transfection. The mLuc-LNPs_ssPalmE-Phe_ exhibited significantly higher luciferase activity in the BMDCs compared to the mLuc-LNPs_ssPalmE_. The gene expression of the mLuc-LNPs_ssPalmE-Phe_ in RAW 264.7 cells was also significantly higher than that of the LNPs_ssPalmE_ (Figure 2c). A similar trend was also found in the in vivo gene expression of mLuc-LNPs on the skin at the back of the neck at a dose of 1.0 µg of mRNA (Figure 2d,e).

**Figure 3:**
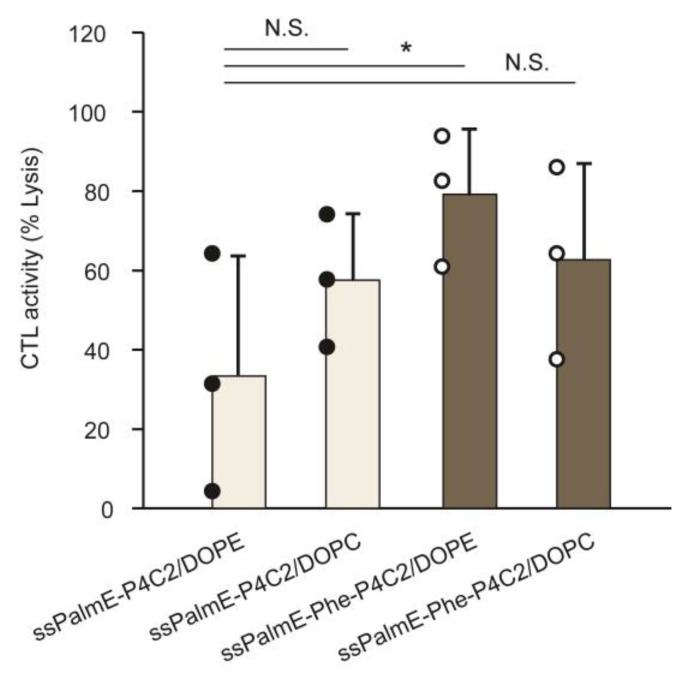
CTL activity of the LNPs_ssPalmE_ and LNPs_ssPalmE-Phe_ prepared using a microfluidic device. The mOVA-LNPs_ssPalmE_ and mOVA-LNPs_ssPalmE-Phe_, with either DOPE or DOPC as helper lipids, were evaluated for their immune activation activity via a CTL assay. The CTL assays of the mOVA-LNPs were conducted in C57BL/6J mice via immunization (s.c.) of 0.05 µg of mRNA. On day 7 after immunization, the mice were injected (i.v.) with equal amounts of CFSE^hi^-labelled splenocytes (SIINFEKL OVA epitope) and CFSE^low^-labelled splenocytes (naïve). The spleen was collected on Day 8, and the % lysis of splenocytes was quantified via flow cytometry. The scatter graph represents individual values; the bar graph represents the mean with SD (*n* = 3). N.S.: not significant; * *p* < 0.05 (one-way ANOVA followed by the Bonferroni test against the original LNP_ssPalmE_).

The ability to activate cell-mediated immunity for both the LNPs_ssPalmE-Phe_ and the LNPs_ssPalmE_, with either DOPE or DOPC as helper lipids, was evaluated with a CTL assay against the model antigen ovalbumin (OVA). As shown in Figure 3, the mOVA-LNPs_ssPalmE-Phe_ had an overall higher CTL activity than the mOVA-LNPs_ssPalmE_, with either helper lipid being used. The use of DOPE as a helper lipid in the mOVA-LNPs_ssPalmE-Phe_ provided the highest CTL activity (Figure 3). Collectively, the higher gene expression activity and vaccination activity of the LNPs_ssPalmE-Phe_ indicated that the insertion of a phenyl ester group improved the function of the ionizable lipid with a vitamin E scaffold.

### 3.2. Ready-to-Use-Type RNA Vaccine Development

The preparation method for the lyophilized ready-to-use LNP formulation (LNP_ssPalm_(RtoU)) was then applied to the LNPs_ssPalmE-Phe_ to further improve their usability. To apply the preparation method, the lipid composition was adjusted to the previously reported LNPs_ssPalm_(RtoU) (ssPalm/DOPC/Chol = 52.5/7.5/40 (% of total lipid)) [17,20]. We initially applied both the default RtoU composition (ssPalmE-Phe-P4C2/helper lipid/cholesterol = 52.5/7.5/40) and the microfluidic-mixer-type LNPs_ssPalmE-Phe_ (ssPalmE-Phe-P4C2/helper lipid/cholesterol = 60/30/10), with a fixed DMG-PEG_2000_ ratio of 3 mol% of total lipid. Two types of helper lipids, DOPE and DOPC, were used in the lipid formulation. A stock solution of the lipids prepared in 90% t-butanol was prepared. The mixture of the lipids was then mixed with a 40 mM MES (pH 5.0) buffer with a NanoAssemblr device to prepare empty LNPs. An equal volume of 320 mg/mL of sucrose was then added into the empty LNPs_ssPalmE-Phe_. The LNP solution was frozen in liquid nitrogen and then transferred to the freeze-drying equipment. The recovered lyophilized empty LNPs_ssPalmE-Phe_(RtoU) can be reconstituted with the intended mRNA solution in an aqueous medium, followed by heating at 95 °C for 5 min. After cooling, the LNPs were neutralized with an equal volume of PBS and used for the following experiments/analysis (Figure 1b).

**Figure 4:**
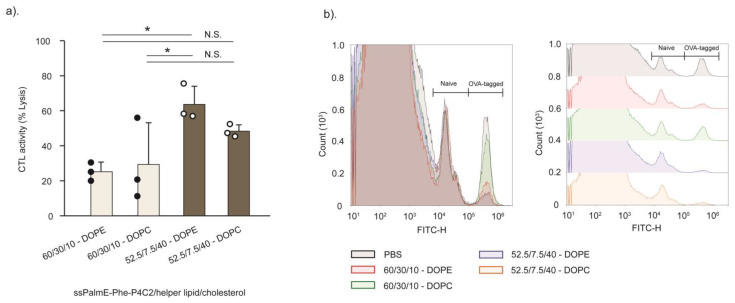
CTL activity of LNPs_ssPalmE-Phe_(RtoU) with different lipid compositions. (**a**) The activation of the antigen-specific cellular immunity of the LNPs_ssPalmE-Phe_(RtoU) with the lipid composition of ssPalmE-Phe-P4C2/(DOPE or DOPC)/cholesterol = 60/30/10 and ssPalmE-Phe-P4C2/(DOPE or DOPC)/cholesterol = 52.5/7.5/40 was evaluated via CTL assays. All lipid compositions included an additional 3 mol% of DMG-PEG_2000_. (**b**) The overlaid (left) and offset (right) FACS histogram from the CTL assay. CTL assays of mOVA-LNPs(RtoU) were conducted in C57BL/6J mice via immunization (s.c.) with 0.1 µg of mRNA. The spleen was collected, and the % lysis of splenocytes was quantified via flow cytometry. The scatter graph represents the individual values; the bar graph represents the mean with SD (*n* = 3). N.S.: not significant; * *p* < 0.05 (one-way ANOVA followed by SNK test).

The physicochemical properties of mOVA-LNPs_ssPalmE-Phe_(RtoU) are shown in Table 1. The mOVA-LNPs_ssPalmE-Phe_(RtoU) with the ssPalmE-Phe-P4C2/helper lipid (DOPE or DOPC)/Chol = 60/30/10 formulation showed particle sizes of around 110–120 nm with a slightly high PdI (0.200–0.240) and a neutral charge (−2 mV). On the other hand, in the case of the other lipid composition, the ssPalmE-Phe-P4C2/helper lipid/Chol = 52.5/7.5/40 formulation, the particle sizes were slightly larger (120–140 nm), with an acceptable range of PdI (≤0.200) and a light negative charge (−10 mV). All formulations had high mRNA encapsulation efficiencies (>90%) after simple rehydration and incubation. The immune activation activity of the LNPs_ssPalmE-Phe_(RtoU) was compared with their ability to induce CTL activity. The reconstituted mRNA-LNPs_ssPalmE-Phe_(RtoU) were directly injected into the mice for immunization. The CTL activity was not affected by the presence or absence of ultrafiltration steps (Figure S1) for the mRNA-LNPs_ssPalmE-Phe_(RtoU). The mOVA-LNPs_ssPalmE-Phe_(RtoU) with the ssPalmE-Phe-P4C2/helper lipid (DOPE or DOPC)/Chol = 52.5/7.5/40 formulations showed an overall better induction of CTL activity (Figure 4). Moreover, the use of DOPE resulted in the highest CTL activity (Figure 4). These results indicate that the LNP_ssPalmE-Phe_(RtoU) with a composition of ssPalm/DOPE/Chol = 52.5/7.5/40 is suitable for the development of an RNA vaccine.

### 3.3. Optimization of Buffer pH and Cryoprotectant Concentration

Concerning the preparation of the LNPs_ssPalmE-Phe_(RtoU), an acidic buffer pH is important since the cationic charge is accompanied by the protonation of the ionizable lipid, which is plausibly a crucial driving force for the post-encapsulation of the negatively charged mRNA. In parallel, a cryoprotectant is needed to protect the LNPs from damage during the freeze-drying process [20]. The regimen for preparing the LNPs_ssPalmE-Phe_(RtoU) was optimized by changing the pH of the 20 mM MES buffer (pH 5.00–6.00) and sucrose concentrations (80–320 mg/mL). As a first screening, the freeze-dried appearance and physicochemical properties of the mOVA-LNPs_ssPalmE-Phe_(RtoU) after rehydration are also shown in Table 2. The appearance of the freeze-dried material was labeled as ‘good’, ‘dent’, ‘crack’, and ‘collapse’ (Figure S2) based on the literature [21,22]. The LNPs_ssPalmE-Phe_(RtoU) that were labeled as ‘good’ or ‘dent’ were considered to have an acceptable appearance [22]. The lyophilized LNPs_ssPalmE-Phe_(RtoU) that showed an acceptable appearance and properties were further evaluated for their capabilities to induce immune activity: LNPs_ssPalmE-Phe_(RtoU) with a buffer of pH 5.25 had 80 mg/mL of sucrose, pH 5.25—160 mg/mL of sucrose, pH 5.50—160 mg/mL of sucrose, and pH 5.00—320 mg/mL of sucrose.

**Figure 5:**
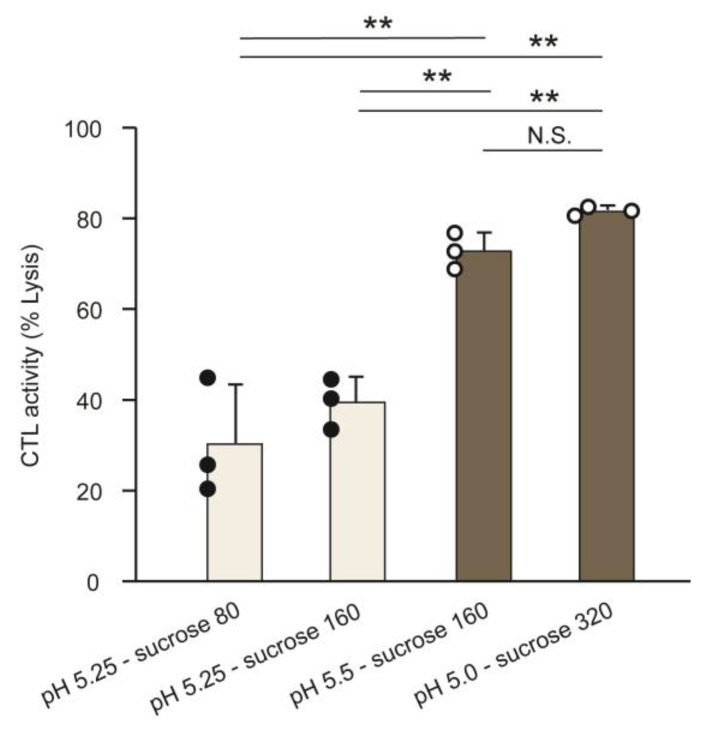
CTL activity of LNPs_ssPalmE-Phe_(RtoU) prepared using different buffer pHs and sucrose concentrations. The CTL activity for combined buffer pH(s) and sucrose concentrations was evaluated and exhibited acceptable appearances and physicochemical properties, as shown in Table 2. A CTL assay of mOVA-LNPs(RtoU) was conducted in C57BL/6J mice via immunization (s.c.) with 0.1 µg of mRNA. The spleen was collected, and the % lysis of splenocytes was quantified via flow cytometry. The scatter graph represents individual values; the bar graph represents the mean with SD (*n* = 3). N.S.: not significant; ** *p* < 0.01 (one-way ANOVA followed by SNK test).

As a result, the LNPs_ssPalmE-Phe_(RtoU) prepared with a buffer pH of 5.00 and 320 mg/mL of sucrose exhibited the highest CTL activity among the other groups and was comparable with those with a buffer pH of 5.50 and a sucrose concentration of 160 mg/mL (Figure 5). The lyophilized appearance of the LNPs_ssPalmE-Phe_(RtoU) with a buffer with a pH of 5.00 and a sucrose concentration of 320 mg/mL (dent) appeared to be not as good as those with a buffer pH of 5.50 and a sucrose concentration of 160 mg/mL (good). However, the particle size and PdI of the LNPs_ssPalmE-Phe_(RtoU) with a buffer pH of 5.00 and a sucrose concentration of 320 mg/mL were better (Table 2). This indicated that the ‘dent’ appearance might have negligible effects on the properties of the of LNPs_ssPalmE-Phe_(RtoU) after hydration with mRNA. Based on these results, we concluded that the optimal buffer and sucrose concentrations for preparing the LNPs_ssPalmE-Phe_(RtoU) were 20 mM of MES buffer (pH 5.00) and 320 mg/mL of sucrose, respectively.

### 3.4. Lipid Composition Screening

**Figure 6:**
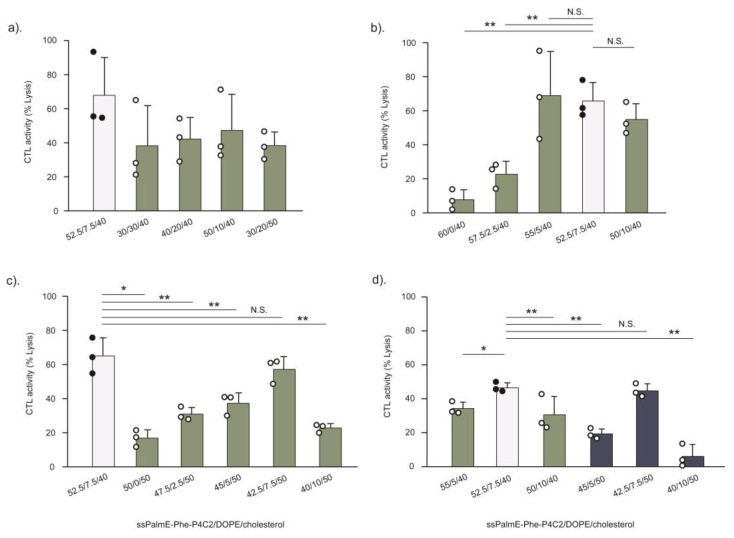
CTL activity evaluation of LNPs_ssPalmE-Phe_(RtoU) prepared using various lipid compositions. The LNPs_ssPalmE-Phe_(RtoU) were prepared with a total of 24 lipid compositions (Table S2), and their immune activation activities were evaluated via CTL assays at a dose of 0.1 µg of mRNA. (**a**) CTL activity of LNPs_ssPalmE-Phe_(RtoU) with 40% Chol. (**b**) CTL activity of LNPs_ssPalmE-Phe_(RtoU) with 0–10% DOPE (with 40% Chol). (**c**) CTL activity of LNPs_ssPalmE-Phe_(RtoU) with 0–10% DOPE (with 50% Chol). (**d**) CTL activity of LNPs_ssPalmE-Phe_(RtoU) with a DOPE ratio of 5–10% and 40 or 50% Chol. The lipid composition of ssPalmE-Phe-P4C2/DOPE/Chol = 52.5/7.5/40 was still found to be the favorable formulation for LNPs_ssPalmE-Phe_(RtoU). The scatter graph represents individual values; the bar graph represents the mean with SD (*n* = 3); white bar: control group; N.S.: not significant; * *p* < 0.05; ** *p* < 0.01 (one-way ANOVA followed by Bonferroni testing against the control group). There were no significant differences found in all groups (against control group) in Figure 6a.

To investigate the issue of whether the ssPalm-Phe-P4C2/DOPE/Chol = 52.5/7.5/40 composition was optimal for LNPs_ssPalmE-Phe_(RtoU), a screening of the lipid composition was further conducted using a total of 24 lipid compositions with various cholesterol and DOPE ratios (Table S2) through CTL assays. All formulations produced mRNA-LNPs_ssPalmE-Phe_(RtoU) with sizes ranging from 160 to 230 nm, PdIs ranging from 0.150 to 0.240, acceptable appearances (dent), encapsulation efficiencies ≥90%, and recovery rates ≥80%. The mRNA-LNPs_ssPalmE-Phe_(RtoU) with the ssPalmE-Phe-P4C2/DOPE/Chol = 52.5/7.5/40 formulation were used as a control in each evaluation. Within the same cholesterol ratio, the CTL activities were maintained or tended to increase with a reduction in the DOPE ratio (Figure 6a and Figure S3a–c). Thus, a lipid composition with a lower DOPE density was evaluated.

In the LNPs_ssPalmE-Phe_(RtoU) with 40% Chol, the optimal range of DOPE was narrow (5–10%): the CTL activity increased when the DOPE ratio was 0–5% but then slightly decreased when the density of DOPE reached 7.5% (Figure 6b). Meanwhile, for the LNPs_ssPalmE-Phe_(RtoU) with 50% Chol, the CTL activity increased when the DOPE ratio was 0–7.5% and then sharply decreased with a DOPE ratio above 10% (Figure 6c). Similarly, the optimal DOPE ratio was also narrow (5–10% DOPE).

Finally, the CTL activities of the LNPs_ssPalmE-Phe_(RtoU) with 5–10% DOPE and 40 or 50% Chol were compared. The LNPs_ssPalmE-Phe_(RtoU) with 50% Chol and a DOPE ratio of 7.5% showed comparable CTL activity with those with 40% Chol and a DOPE ratio of 7.5% (control) (Figure 6d). However, the CTL activity of the LNPs_ssPalmE-Phe_(RtoU) with 50% Chol was more sensitive to the content of DOPE (Figure 6d). This drastic loss in CTL activity depending on the DOPE density is not desirable when considering batch-to-batch reproducibility. We therefore concluded that the initial formulation of ssPalmE-Phe-P4C2/DOPE/Chol = 52.5/7.5/40 was the most favorable lipid composition for LNPs_ssPalmE-Phe_(RtoU) from the viewpoint of CTL activity and physicochemical properties: with a particle size of 158.9 ± 0.4 nm, a PdI of 0.19 ± 7.8, a dented freeze-dried appearance, a 100 ± 2.3% encapsulation efficiency, and a 102.1 ± 7.1% recovery rate.

### 3.5. Therapeutic Anti-Tumor Response against E.G7-OVA Tumor Model

**Figure 7:**
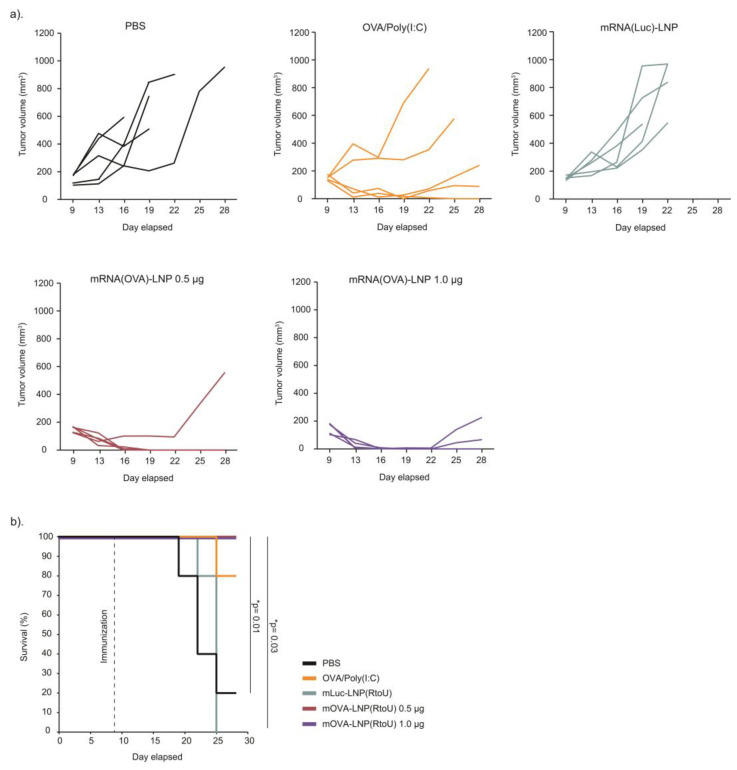
Therapeutic anti-tumor response of LNPs_ssPalmE-Phe_(RtoU). The antitumor response of LNPs_ssPalmE-Phe_(RtoU) against E.G7-OVA tumor-bearing mice was evaluated. E.G7-OVA cells (8.0 × 10^5^ cells/40 µL) were s.c. inoculated on the left flank of C57BL/6J mice (*n* = 5). After the tumor grew to ≥100 mm^3^ in volume, mOVA-LNP_ssPalmE-Phe_(RtoU) was administered (s.c.) at a dose of 0.5 or 1.0 µg of mRNA. The tumor volumes were monitored in 3-day intervals from day 9 to day 25. The end point was set at a tumor volume of 1000 mm^3^. (**a**) Individual tumor growth in each treatment group. (**b**) The overall survival of tumor-bearing mice was determined through a Kaplan–Meier analysis. Significant differences were found between mOVA-LNPs(RtoU), 0.5 µg and 1.0 µg, with PBS (* *p* = 0.01) and mLuc-LNPs(RtoU) (* *p* = 0.03).

To determine the capability of the LNP_ssPalmE-Phe_(RtoU) to function as an mRNA-based vaccine carrier, its therapeutic anti-tumor response against an E.G7-OVA (murine-thymus-lymphoma-expressing ovalbumin) tumor model was evaluated. A subcutaneous administration (a single dose of 0.5 µg of mRNA) significantly suppressed tumor progression. Moreover, the survival analysis indicated that the immunized mice completely survived until 30 days after the tumor inoculation, when the endpoint was set at a 1000 mm^3^ tumor size (Figure 7). Collectively, LNPs_ssPalmE-Phe_(RtoU) can be a potent mRNA-based vaccine carrier.

## 4. Discussion

The ionizable lipid ssPalmE-Phe-P4C2 was designed so as to combine two important functions for an RNA vaccine: a vitamin E scaffold for immune activation and a phenyl ester for self-degradability, respectively. Regarding Vitamin E, it has been used as a key component in AS03, a squalene-based oil-in-water emulsion adjuvant that is used in vaccines against the avian influenza virus [23,24,25]. It has been reported that the vitamin E in the AS03-adjuvanted vaccine modulates the production of cytokines and chemokines such as CCL2, CCL3, IL-6, CSF3, and CXCL1 and promotes antigen-loading monocytes as well as the recruitment of granulocytes [26]. The ssPalm with vitamin E scaffolds (ssPalmE) was initially developed to deliver nucleic acids to the liver; the delivery of siRNA by LNPs_ssPalmE_ demonstrated gene knockdown efficiency and extensive liver accumulation compared to other ssPalms with myristic acid or retinoic acid [15]. The molecular tuning of the ssPalmE through attaching tertiary amines to a piperizine ring resulted in the formation of the second-generation ssPalmE-P4C2, which demonstrated an efficient endosomal escape activity, which contributed to the improved gene knockdown efficiency [15,17]. However, LNPs prepared with ssPalmE derivatives (with piperazine as the tertiary amine structure) triggered severe inflammatory responses that involved the production of pro-inflammatory cytokines (IL6, TNFα), the intracellular DNA-sensor-related cytokine (IL-1β), and interferon (IFNβ) when they were combined with plasmid DNA. From these observations, we hypothesized that the LNPs formed with the ssPalmE derivative would be applicable for use as an RNA vaccine [16]. It was revealed that the mRNA LNPs_ssPalmE_ acted as both a nucleic acid (mRNA) carrier and as an immune adjuvant by inducing the proliferation of antigen-specific CD8+ T cells and their differentiation into effector and memory cells to activate cellular immunity [18]. However, the mechanism concerning the ssPalmE-triggered immune activation is not clear.

Studies have demonstrated the capability of vaccines and an adjuvant to induce immunogenicity by stimulating the damage-associated molecular patterns (DAMPs) pathway [27,28]. Cellular stressors or cell death signaling can trigger the release of DAMPs that are then recognized by other cells via pattern recognition receptors (PRRs). This interaction will result in immunogenic reactions, such as the upregulation of the chemokine/cytokines involved in immune responses. Renowned vaccine adjuvants such as Alum, AS03, and HP-β-cyclodextrin are known to induce the release of DAMP signals, which stimulates and enhances the immunogenicity of vaccines [27]. Our previous study also reported the detection of dsDNA at the injection site (skin) upon LNP_ssPalmE_ administration. This finding suggests that the adjuvant effect of LNPs_ssPalmE-Phe_(RtoU) could also be dependent on this DAMP pathway [18]. Further investigation is required into the cells and molecules involved in this response. It is noteworthy that the use of a simple combination of α-tocopherol and α-tocopherol succinates as a component of poorly immune-stimulative LNPs (formed with a myristic acid-scaffold ssPalm) did not enhance cytokine production [18]. The covalent linkage of α-tocopherol and a tertiary amine into a single molecular structure and/or the dimerization of α-tocopherol could be key factors in the adjuvant effect. Thus, it is plausible that the action of the LNPs_ssPalmE_ involves a mechanism that differs from that of AS03.

The other molecular design integrated in ssPalmE-P4C2 is the introduction of a phenyl ester. The degradability of ionizable lipids in the body has been investigated as a strategy for reducing adverse side effects caused by the accumulation of lipid-like materials. The introduction of ester bonds and/or disulfide bonds is one of the common strategies for achieving the biodegradation of an LNP [15]. It was reported that the combination of disulfide bonds and a phenyl ester moiety synergistically enhances the transfection activity of mRNA by inducing an intraparticle self-degradation that occurs within the limited intraparticle spaces. This reaction induces the release of the nucleic acid cargo [17]. Actually, an siRNA-LNP that contains an oleic-acid-based self-degradable ssPalm (ssPalmO-Phe-P4C2) showed an ED_50_ of 0.0044 mg/kg siRNA in the liver when administered via an i.v. injection [15]. Comparing the knockdown activity of ssPalmE-P4C2 with that of ssPalmO-Phe-P4C2, the findings suggested that the efficiencies of nucleic acid delivery for these two materials were significantly different. From this finding, we concluded that the combination of vitamin E scaffolds and phenyl esters is a strategy that can satisfy both immunostimulatory properties and nucleic acid delivery efficiency. The improvement in the luciferase activity and CTL activity of LNP_ssPalmE-Phe_ over LNP_ssPalmE_ suggested that the release of mRNA promoted by self-degradability is an important factor for improving the efficacy of an RNA vaccine. The LNP_ssPalmE-Phe_(RtoU) also demonstrated strong CTL activity and a significant suppression of E.G7-OVA tumor growth (Figure 7). These observations suggest that ssPalmE-Phe-P4C2 would be a reasonable candidate for use in designing an RNA vaccine that focuses on the activation of cellular immunity.

It is noteworthy that the mRNAs used in this study were unmodified/unpurified. However, in the case of ionizable lipids with vitamin E scaffolds, the hydrophobic scaffold contributed significantly to the adjuvant activity of the LNPs [18]. The contribution of mRNA to the adjuvant activity was negligible. Therefore, ssPalmE-Phe-P4C2, rather than the IVT-mRNA, is considered to be responsible for the immunostimulatory properties of the mRNA-LNPs [18]. Since the improvement in the quality of mRNA can positively affect the vaccine activity of an RNA vaccine [29], the combination of chemically modified and purified mRNA with LNPs_ssPalmE-Phe_(RtoU) would be a potent strategy for the further development of cancer vaccines.

The ready-to-use (RtoU) formulation, a freeze-drying-based preparation method of mRNA-LNPs, was then applied to the LNPs_ssPalmE-Phe_ to further develop them as a convenient nucleic acid carrier. Freeze-drying has been extensively used in pharmaceutical industries to improve the stability and shelf life of drug products [19,30,31]. However, the stresses associated with freezing and drying can damage biomolecules unless an appropriate cryoprotectant and lipid composition are used [32]. In this study, the successful application of an RtoU formulation produced mRNA-LNPs that allow for the post-encapsulation of the desired nucleic acids to be achieved via simple rehydration and incubation. The LNP_ssPalmE-Phe_(RtoU) offers handling practicality and a high rate of encapsulation (>90%) with a minimum loss of nucleic acids. The limitation of the LNP_ssPalmE-Phe_(RtoU) formulation involves the concentration (1.0 µg mRNA/200 µL) and scale (1.0 µg mRNA/vial) of the resultant mRNA-LNPs. Although this is sufficient for the vaccine experiment in mice, this point should be improved from the viewpoint of other applications.

Sucrose acts as a cryoprotectant, which helps to maintain the stability and integrity of LNPs during the freeze-drying process. The Pfizer/BioNTech and Moderna COVID-19 mRNA vaccines both used sucrose as a cryoprotectant to protect and stabilize the LNPs while in a deep-frozen state (−80 °C) during distribution [33,34,35,36]. The Food and Drug Administration authorized the most recent formula of the Pfizer/BioNTech and Moderna COVID-19 mRNA vaccines (2023–2024 Formula), which corresponds to the Omicron variant XBB.1.5 of SARS-CoV-2. In this recent formula, the Pfizer/BioNTech COVID-19 mRNA vaccine used 31 mg of sucrose in each 0.3 mL dose (103.3 mg/mL) [37]; meanwhile, the Moderna COVID-19 mRNA vaccine used 21.8 mg of sucrose in each 0.25 mL dose (87.2 mg/mL) [38]. Compared to a frozen vaccine product, such as the COVID-19 mRNA vaccines, a much higher sucrose concentration is needed to stabilize a lyophilized vaccine product. It was mentioned that sugar molecules replace the water between the hydrophilic heads of phospholipids during lyophilization, thus lowering the phase transition temperature and preventing a gel-to-liquid phase transition [31]. It was also suggested that sugar forms glass matrixes, trapping phospholipids upon the removal of water, which prevents lipid aggregation and damage by ice crystals [31,39]. In this study, sucrose was used as a cryoprotectant for the LNPs_ssPalmE-Phe_(RtoU) following a previously reported procedure [20,40]. The dent or shrinkage appearance of the LNPs_ssPalmE-Phe_(RtoU) is probably related to the formulation process, in which the secondary drying process was apparently too rapid [22]. The amount of sucrose and/or the freeze-drying process should be optimized for each application. In our experiments, the dent appearance of the lyophilized product had no effect on the product’s quality. This is consistent with the general notion that a dent appearance is acceptable [22]. The pH of the buffer is another important factor in preparing an LNP, especially for the post-encapsulation of mRNA. The electrostatic interaction between ionizable lipids and nucleic acids is probably the main driving force in the post-encapsulation process [20]. It should be noted here that the buffer pH (5.0) together with a high concentration of sucrose (320 mg/mL) produced preferable particle properties (Table 2) and a high immune activation activity (Figure 5).

The established RtoU formulation (ssPalm/helper lipid/cholesterol = 52.5/7.5/40) [20] was found to be the most suitable lipid composition for the LNPs_ssPalmE-Phe_, which demonstrated stronger immune activation activity compared to the other formulations (Figure 6). This observation is closely related to the cholesterol composition. Cholesterol is known to have the ability to provide cryoprotection for LNPs during freeze-drying by maintaining the stability of lipid formation and preventing the risk of leakage of contents [41,42]. In addition, it was revealed that a cholesterol content of at least 40% in the LNPs_ssPalm_ was essential for achieving a high transfection activity of the phenyl-type ionizable lipid [17]. The ONPATTRO^®^ or Patisiran (Alnylam Pharmaceutical, Cambridge, MA, USA) also utilizes a cholesterol content > 30% (38.5%) in its LNP formulation for siRNA delivery [33]. Moreover, the current COVID-19 mRNA vaccines by Pfizer/BioNTech and Moderna also utilize a cholesterol content >30% in their LNP formulation, with 42.7% and 38.5% cholesterol, respectively [33]. Although the exact composition of cholesterol varies depending on the type of ionizable lipid and its delivery purpose, a cholesterol content of at least around 40% in the LNP formulation might be important in terms of exerting its mRNA delivery efficiency. The limitation of the LNP(RtoU) formulation is the concentration (1.0 µg/200 µL) and scale (1.0 µ/vial) of the resultant mRNA-LNPs. Although this is enough for the vaccine experiment in mice, this point should be improved from the viewpoint of other applications.

Phospholipids are also included as helper lipids to provide particle stability and delivery efficiency [43]. DOPE is a cone-shaped lipid with high fusogenic activity, while DOPC functions to stabilize the LNPs through its overall cylindrical shape. Both lipids were used to form the microfluidic-mixer-type (Figure 3) or the RtoU-type (Figure 4) LNPs_ssPalmE-Phe_. As a result, the use of DOPE was revealed to be important for achieving higher activity of cytotoxic T cells. The findings indicated that the CTL activity of the LNPs_ssPalmE-Phe_(RtoU) tended to increase when the DOPE ratio was reduced. However, the complete removal of the helper lipid was shown to be detrimental to the CTL activity of the LNPs_ssPalmE-Phe_(RtoU). This indicates there is a delicate balance between the ionizable lipid, the helper lipid, and the amount of cholesterol needed to achieve an optimum lipid composition. When the cholesterol ratio was fixed, the DOPE ratio was accompanied by an increase in the ssPalmE-Phe-P4C2 ratio. Thus, the amount of ionizable lipid holds priority in the lipid composition, which determines the immune activation activity of the LNPs_ssPalmE-Phe_(RtoU).

## 5. Conclusions

Ready-to-use-type LNPs_ssPalmE-Phe_ offer a convenient form of an mRNA-based vaccine carrier. The LNPs_ssPalmE-Phe_(RtoU) were capable of post-encapsulating the intended mRNA antigens through a simple rehydration/incubation step. The efficacy of the LNPs_ssPalmE-Phe_(RtoU) to significantly suppress tumor progression indicated their usefulness in cancer vaccine development. Therefore, this RtoU technology would be suitable for easily screening novel antigens/neo-antigens and/or confirming a proof-of-concept as to whether the candidate gene actually functions as an antigen for cancer therapy.

## Figures and Tables

**Table 1:** Physicochemical properties of LNPs_ssPalmE-Phe_(RtoU) with different compositions.

Composition (%)	Helper Lipid	mRNA	Size (d.nm)	PdI	ZP	Encaps.	Recovery
					(mV)	(%)	(%)
60/30/10	DOPE	empty	115.6	0.18	−1.2	-	-
60/30/10	DOPE	OVA	113.4	0.22	−2.8	94.3	83.2
60/30/10	DOPC	OVA	117.9	0.24	0.7	93.7	99.1
52.5/7.5/40	DOPE	empty	130.2	0.17	−8.3	-	-
52.5/7.5/40	DOPE	OVA	128.1	0.18	−10.7	94.3	77.0
52.5/7.5/40	DOPC	OVA	131.4	0.19	−9.2	94.3	81.7

Size, PdI, and ZP were measured with Zetasizer Nano ZS. Encapsulation efficiency was measured with Ribogreen^®^ assay. Composition (%) represents the ssPalmE-Phe-P4C2/helper lipid/cholesterol ratio.

**Table 2:** Physicochemical properties of the LNPs_ssPalmE-Phe_(RtoU) prepared with different buffer pH(s) and sucrose concentrations.

Sucrose (mg/mL)		Buffer pH	Size (d.nm)	PdI	ZP (mV)	Appearance	Encaps. (%)
80		5.0	167.2	0.16	−10.6	good	82.8
	*	5.25	153.7	0.10	−11.5	good	99.4
		5.50	179.8	0.17	−11.5	crack	100.7
		5.75	220.5	0.32	−12.3	crack	101.1
		6.0	303.2	0.48	−12.6	crack	100.8
160		5.0	170.2	0.14	−13.0	good	100.6
	*	5.25	169.4	0.13	−11.1	good	99.3
	*	5.50	206.6	0.22	−11.8	good	100.6
		5.75	244.2	0.34	−12.6	good	100.8
		6.0	287.9	0.40	−13.2	good	100.4
320	*	5.0	160.9	0.19	−12.9	dent	95.5
		5.25	311.1	0.26	−13.1	dent	100.2
		5.50	417.4	0.69	−13.3	dent	100.0
		5.75	313.2	0.42	−14.3	dent	100.4
		6.0	1158.0	0.79	−14.5	dent	98.3

Size, PdI, and ZP were measured with Zetasizer Nano ZS. Encapsulation efficiency was measured with Ribogreen^®^ assay. The samples with asterisks (*) were evaluated for CTL activity.

## Data Availability

The data presented in this study are available on request from the corresponding author.

## Supplementary Materials

The following supporting information can be downloaded at https://www.mdpi.com/article/10.3390/pharmaceutics15122702/s1: Table S1: Detailed list of materials used in the study; Table S2: Physicochemical properties of LNP_ssPalmE-Phe_(RtoU) lipid composition screening; Figure S1: CTL activity of LNPs_ssPalmE-Phe_(RtoU) prepared with or without ultrafiltration; Figure S2: Freeze-dried appearance of LNPs_ssPalmE-Phe_(RtoU); and Figure S3: CTL activity of LNPs_ssPalmE-Phe_(RtoU) prepared with various lipid compositions.
