# Supplementary material for: Development of a Ready-to-Use-Type RNA Vaccine Carrier Based on an Intracellular Environment-Responsive Lipid-like Material with Immune-Activating Vitamin E Scaffolds
Source: Pharmaceutics. 2023 Nov 29;15(12):2702. doi: 10.3390/pharmaceutics15122702 (PMC10747879; doi:10.3390/pharmaceutics15122702)
Supplement: Supplementary file 1 [file pharmaceutics-15-02702-s001.zip › pharmaceutics-2694644-supplementary.pdf]

pharmaceutics

Supplementary Materials

Development of a Ready-to-Use-Type RNA Vaccine Carrier Based on An Intracellular Environment-Responsive Lipid-Like Material with Immune-Activating Vitamin E Scaffolds

Jessica Anindita[1,2†], Hiroki Tanaka[2*†], Ryotaro Oyama[1,2], Shinya Hagiwara[1], Daiki Shirane[1], Sakurai Taneichi[3], Yuta Nakai[3], Kota Tange[3], Hiroto Hatakeyama[1], Yu Sakurai[2], Hidetaka Akita[2*]

[1] Laboratory of DDS Design and Drug Disposition, Graduate School of Pharmaceutical Sciences, Chiba University, 1-8-1 Inohana, Chuo-ku, Chiba, 260-0856, Japan

[2] Laboratory of DDS Design and Drug Disposition, Graduate School of Pharmaceutical Sciences, Tohoku University, 6-3 Aoba, Aramaki, Aoba-ku, Sendai, 980-8578, Japan

[3] Life Science Research Laboratory, NOF CORPORATION, 3-3 Chidori-cho, Kawasaki-ku, Kanagawa, 210-0865, Japan

[†]These authors contributed equally to this study

*Corresponding author:
email address: hiroki.tanaka.e1@tohoku.ac.jp (H. Tanaka)
Tel.: +81-22-795-6831
email address: hidetaka.akita.e4@tohoku.ac.jp (H. Akita)
Tel.: +81-22-795-6831

pharmaceutics

MDPI

Supplementary Table S1. Detailed list of materials used in the study

Reagent	Size	Manufacturer	Product number
ssPalmE-P4C2 (COATSOME®SS-EC)	1 g	NOF	SS-EC
ssPalmE-Phe-P4C2	300 mg	NOF	SS-EP
DOPE; 1,2-dioleoyl-*sn*-glycero-3-phos-phoethanolamine)	500 mg	Avanti Polar Lipids	850725p-500mg
COATSOME®MC-8181 (DOPC; 1,2-di-oleoyl-*sn*-glycero-3-phosphocholine)	1 g	NOF	302-16881
Cholesterol	5 g	Sigma	C8667-5G
DMG-PEG2000 (SUNBRIGHT®GM-020) (1,2-dimyristoyl-rac-glycero-3-methoxypolyethylene glycol) 2000	1 g	NOF	GM-020
Sucrose	500 g	nacalai tesque	30404-45
DL-Malic Acid	500 g	nacalai tesque	21029-55
Sodium Chloride	500 g	nacalai tesque	31320-05
MES (2-(*N*-morpholino)ethanesulfonic acid)	100 g	nacalai tesque	02442-44
HEPES (4-(2-hydroxyethyl)-1-pipera-zineethanesulfonic acid)	500 g	Dojindo	342-01375
TritonX-100 (polyoxyethylene (10) oc-tophenyl ether)	500 mL	FUJIFILM Wako	168-11805
Quant-IT™ RiboGreen® RNA reagent	1 mL	Invitrogen	R11491
D-PBS (-)	500 mL	nacalai tesque	14249-24
99.5% Ethanol	500 mL	nacalai tesque	14713-95
99.5% Ethanol (for Molecular Biology)	500 mL	nacalai tesque	08948-25
t-Butyl Alcohol	500 mL	nacalai tesque	11714-75
RNase Quiet	475 mL	nacalai tesque	09477-94
NucleoSpin Plasmid QuickPure	50 reac-tions	MACHEREY-NAGEL	740615
PCI (phenol: choloroform: isoamyl al-cohol = 25: 24: 1)	100 mL	nacalai tesque	25970-14
20 mg/mL Glycogen	1 mL	nacalai tesque	17110-11
UltraPure ™ DNase/RNase-Free Dis-tilled Water (DDW)	500 mL	Invitrogen	10977-023
XhoI	5000 U	NEB	R0146S
BspEI	1000 U	NEB	R0540S
CutSmart Buffer	5 mL	NEB	B7204S
mMESSAGE mMACHINE® T7 ULTRA Transcription kit	50 reac-tions	Invitrogen	AM1345
THE RNA Storage Solution	1 mL × 10	Invitrogen	AM7000
RPMI 1640	500 mL	Sigma	R8758

FBS (Fetal Bovine Serum)	500 mL	Gibco	10270
FCS (Fetal Calf Serum)	500 mL	Hyclone	SH30910.03
100 mM Sodium Pyruvate	100 mL	nacalai tesque	06977-34
1 mol/l HEPES	100 mL	nacalai tesque	17557-94
55 mM 2-Mercaptoethanol	50 mL	Thermo Fisher	21985023
Penicillin/Streptomycin	100 mL	nacalai tesque	26253-84
Recombinant Mouse GM-CSF	50 µg	R&D SYSTEMS	415-ML-050
G418 Sulfate	5 g	FUJIFILM Wako	074-05963
0.25% Trypsin/EDTA	500 mL	nacalai tesque	32777-15
Red Blood Cell Lysing Buffer	100 mL	Sigma	R7757-100ML
Cellstain CFSE	1 mg	Dojindo	C375
OVA H-2K^b cytotoxic T-lymphocyte epitope peptide (SIINFEKL, OVA$_{257-264}$)	100 mg	TORAY	Customized product
Dulbecco PBS (-)	100 g	Nissui	05913
Albumin, Bovine Serum, General Grade, pH 7.0	100 g	nacalai tesque	01860-07
Sodium Azide	500 mg	FUJIFILM Wako	194-01275
Poly(I:C) H.M.W	10 mg	Invivogen	tlrl-pic
Diethyl ether	500 mL	nacalai tesque	15402-35
D-Luciferin Potassium Salt	1 g	FUJIFILM Wako	126-05116
Reporter Lysis 5× Buffer	30 mL	Promega	E397A
Nano-Glo® Luciferase Assay System	10 mL	Promega	E1501
BCA Protein Assay kit	500 reactions	Takara	T9300A
AmiconUltra-4-100K Centrifugal Units	4 mL volume	Merck	UFC810096
AmiconUltra-4-100K Centrifugal Units	15 mL volume	Merck	UFC910096

pharmaceutics MDPI

Supplementary Table S2. Physicochemical properties of the LNP$_{ssPalmE\text{-}Phe}$(RtoU) lipid composition screening

No.	Variables	Lipid composition (%)			Size (d.nm)	PdI	ZP (mV)	Encapsulation (%)	Recovery (%)
		E-Phe	DOPE	Chol					
1	Chol 10%	30	60	10	172.0	0.20	-2.2	88.7	99.8
2		40	50	10	177.4	0.21	-2.8	89.9	106.7
3		50	40	10	192.1	0.20	-2.9	90.0	99.9
4		60	30	10	187.6	0.21	-3.2	85.2	100.9
5	Chol 20%	30	50	20	176.9	0.23	-2.6	94.7	107.4
6		40	40	20	168.1	0.18	-6.7	96.0	105.0
7		50	30	20	162.3	0.19	-5.5	96.4	104.6
8		60	20	20	160.7	0.21	-7.4	95.7	102.1
9	Chol 30%	30	40	30	170.8	0.24	-7.0	102.3	110.2
10		40	30	30	162.5	0.22	-7.4	104.0	105.7
11		50	20	30	163.3	0.20	-8.6	103.9	105.3
12		60	10	30	165.7	0.22	-9.1	101.2	107.3
13	Chol 40%	30	30	40	167.9	0.22	-7.5	100.3	96.3
14		40	20	40	177.0	0.24	-12.1	99.9	95.8
15		50	10	40	174.7	0.18	-10.6	101.3	101.9
16	Chol 50%	30	20	50	184.5	0.16	-10.8	100.1	99.8
17	Chol 40%; DOPE 2.5-10%	57.5	2.5	40	237.9	0.13	-10.0	99.0	103.0
18		55	5	40	204.2	0.18	-11.3	100.7	122.9
19		52.5	7.5	40	168.4	0.19	-12.5	96.9	115.6
20		50	10	40	175.2	0.20	-12.1	97.7	120.5
21	Chol 50%; DOPE 2.5-10%	47.5	2.5	50	254.5	0.20	-12.5	98.8	116.2
22		45	5	50	161.8	0.16	-11.6	96.5	109.2
23		42.5	7.5	50	207.0	0.19	-14.0	97.6	111.5
24		40	10	50	193.5	0.15	-13.0	98.5	107.9

All compositions produced LNP$_{ssPalmE\text{-}Phe}$(RtoU) with ‘dented’ appearance.

pharmaceutics

MDPI

Supplementary Figure S1. CTL activity of LNPs$_{\text{ssPalmE-Phe}}$(RtoU) prepared with or without ultrafiltration.

The reconstitution step for the freeze-dried LNP$_{\text{ssPalmE-Phe}}$(RtoU) with mRNA in aqueous solution was also optimized so as to provide further flexibility in drug preparation. After reconstitution, the residual sucrose was first removed by ultrafiltration, but this prove to not be practical and time-consuming. Therefore, we eliminated the ultrafiltration step. The vaccination activity of mRNA-LNP(RtoU) was not significantly altered even in the absence of the ultrafiltration step. Thus, the ultrafiltration in the reconstitution step was eliminated and replaced with a simpler mRNA-LNP(RtoU) preparation.

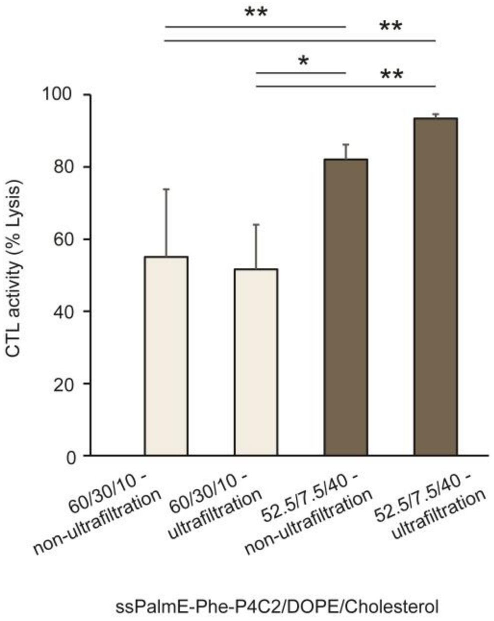

Figure S1. CTL activity of LNPs$_{\text{ssPalmE-Phe}}$(RtoU) LNP with different lipid compositions prepared with or without ultrafiltration. CTL assay of mOVA-LNP(RtoU) was conducted in C57BL/6J mice by immunization (s.c.) with 0.1 µg of mRNA. The spleen was collected and the % lysis of splenocytes was quantified by flow cytometry. Mean with SD (n = 3), *p<0.05, **p<0.01 (one-way ANOVA followed by SNK test).

pharmaceutics
MDPI

Supplementary Figure S2. Freeze-dried appearance of LNPs$_{\text{ssPalmE-Phe}}$(RtoU)

After recovering the freeze-dried LNP$_{\text{ssPalmE-Phe}}$(RtoU)(s), the appearances were checked and the samples evaluated for visible cracks, dents, and collapses in front of a black background. The physical appearances of LNP$_{\text{ssPalmE-Phe}}$(RtoU) were labeled as 'good', 'dent', 'crack', or 'collapse'. The LNP$_{\text{ssPalmE-Phe}}$(RtoU) labeled as 'good' or 'dent' were considered to have an acceptable appearance. The LNP$_{\text{ssPalmE-Phe}}$(RtoU) labeled as 'crack' indicated that the cake was brittle and that a hard solid appearance would not be maintained for a long period of time. Whereas LNP$_{\text{ssPalmE-Phe}}$(RtoU) which labelled as 'collapse' indicated that the LNP formulation failed to form a dry particle after freeze-drying.

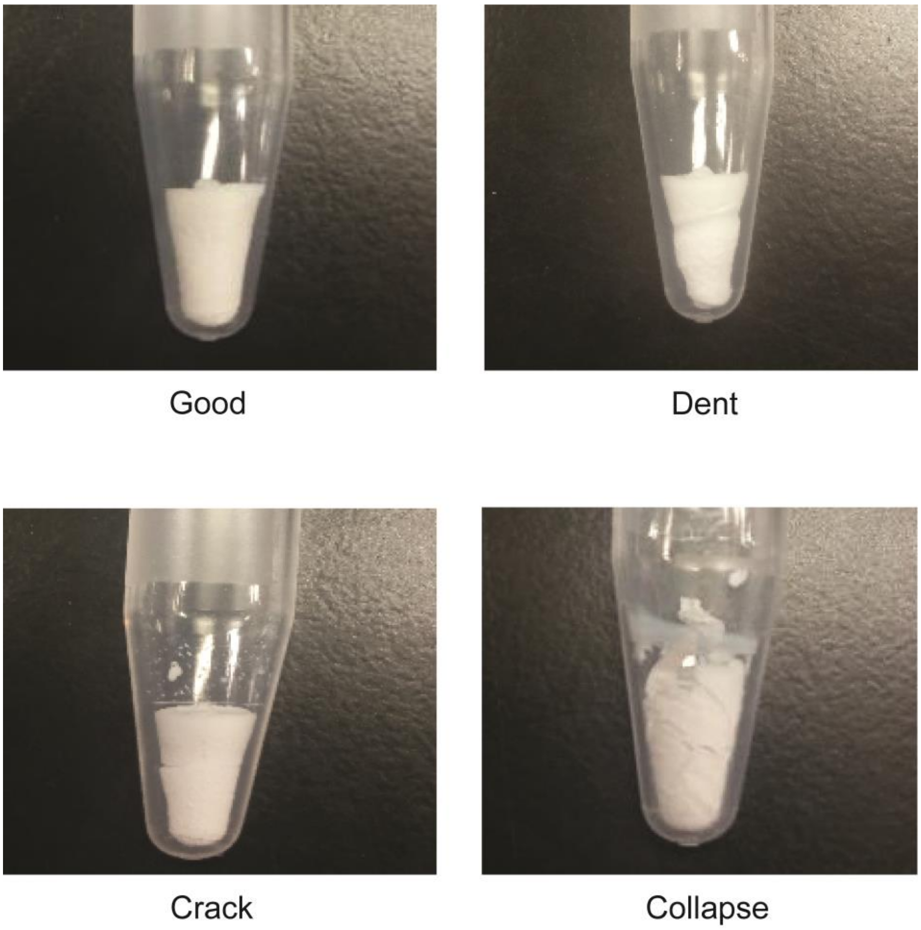

Figure S2. Freeze-dried appearance of LNPs$_{\text{ssPalmE-Phe}}$(RtoU). The appearance of LNP$_{\text{ssPalmE-Phe}}$(RtoU) was defined as good, dent, crack, or collapse (freeze-drying failure).

pharmaceutics

MDPI

Supplementary Figure S3. CTL activity of LNPs$_{ssPalmE\text{-}Phe}$(RtoU) prepared by various lipid compositions.

The lipid composition of the LNP$_{ssPalmE\text{-}Phe}$(RtoU) was evaluated by changing the cholesterol and DOPE ratio. A total of 24 lipid compositions were evaluated (**Supplementary Table S2**) and their physicochemical properties and CTL activity were evaluated. The CTL assay results of lipid compositions with 10%, 20%, and 30% cholesterol ratio are shown in this section. Within the same cholesterol ratio (10%, 20%, or 30%), the CTL activity tended to increase by the reduction of the DOPE ratio.

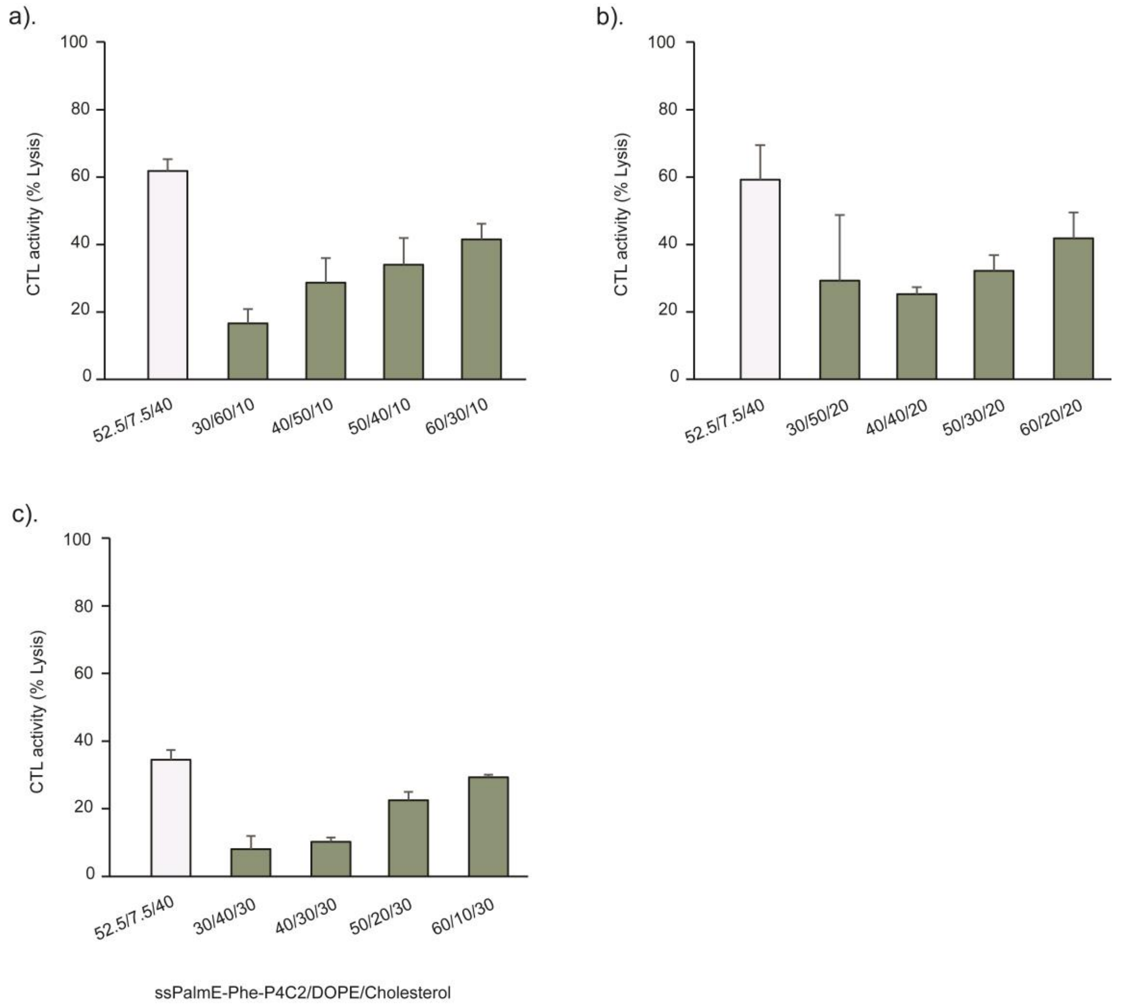

Figure S3. CTL activity of LNPs$_{ssPalmE\text{-}Phe}$.(RtoU) prepared by lipid compositions with fixed 10-30% cholesterol ratios. The ssPalmE-Phe-P4C2 ratio in mOVA-LNP(RtoU) changed depending on the cholesterol and DOPE ratio. **(a)** Fixed Chol: 10%. **(b)** Fixed Chol: 20%. **(c)** Fixed Chol: 30%. CTL assay was conducted in C57BL/6J mice by immunization (s.c.) of 0.1 µg mRNA. The spleen was collected and the % lysis of splenocytes was quantified by flow cytometry. Mean with SD (n = 3), white bar: control group.
